# Outlier data in volume calculations of uterine fibroids comparing ellipsoid formula and voxel-based segmentation

**DOI:** 10.1186/s12880-025-01672-7

**Published:** 2025-05-16

**Authors:** Viktor Bérczi, Kolos György Turtóczki, Szuzina Fazekas, Anna Dolla-Takács, Róbert Stollmayer, Pál Novák Kaposi, Ildikó Kalina, Bettina Katalin Budai

**Affiliations:** Medical Imaging Centre, Semmelweis University, 2 Korányi Sándor St, Budapest, H-1083 Hungary

**Keywords:** Uterine fibroid, Volumetry, Ellipsoid formula, Magnetic resonance imaging, MRI

## Abstract

**Background:**

The ellipsoidal formula is the most common method used to determine the volume of fibroids on MR images. Labor-intensive manual segmentation provides the opportunity to measure the volume of a given lesion on a voxel basis. The aim of this study is to compare the volume of the uterine fibroid calculated using voxel-based segmentation and the ellipsoid formula.

**Methods:**

In this study, pretreatment MRI scans of patients who underwent uterine artery embolization due to symptomatic fibroids were retrospectively collected between 2016 and 2022. The volume data of the largest fibroids was determined by segmentation (group S) as the reference standard. In addition, the largest diameters of the fibroids in three planes (D1/D2/D3) were also measured and the volumes were also estimated by using the ellipsoidal formula (D1*D2*D3*0.5233) (group E). The interobserver reproducibility of the diameter measurements was tested. The volume values (median, IQR) were compared; in addition, the differences between the segmented and ellipsoidal volumes were recorded. Statistical analysis was performed using the Kruskal-Wallis test, Wilcoxon’s two-sided signed rank test, intraclass correlation (ICC) analysis, and Bland-Altman plots.

**Results:**

Pretreatment MRI scans of 113 patients were identified. Fibroids where the interobserver difference of diameter-based ellipsoidal volumes reached 30% were excluded resulting in 99 patients in the final dataset. The volumes of group S and group E showed no significant differences with 134.1 (257.3) cm^3^ and 133.5 (269.1) cm^3^, respectively, with an average difference of 3.47 cm^3^ (0.25%; *p* = 0.377). The agreement between the two methods was excellent (ICC = 0.979), without difference across fibroid locations. In 46 cases (46.5%), group S values were larger, and in 53 fibroids (53.5%), group E volume values were larger. However, volume difference was outside the ± 20% range in 21 cases (21.2%) and outside the ± 30% range in 10 cases (10.1%); the largest difference was approximately 56.5% (156.5 cm^3^).

**Conclusions:**

The ellipsoid formula-based and the voxel-based volume calculation showed no significant difference for the group as a whole. However, there was a difference of > 20% in 21.2% of cases and > 30% in 10.1% of cases. In the era of personalized medicine, it is not only the average difference between the two methods that need to be considered but also cases where there is a 20% or 30% difference in results should be highlighted, as these may change the treatment plan in individual cases. This methodology should also be tested for other tumor-type volume calculations.

## Introduction

The uterine fibroid, also known as leiomyoma, is a benign tumor originating from the smooth muscle cells of the uterus and represents the most common gynecological condition. It can affect 40–70% of women of reproductive age. While fibroids are often asymptomatic, they can cause significant complaints in affected patients. These symptoms include abnormal uterine bleeding, pelvic pain, frequent urination, constipation, back pain, and pain in the legs and abdomen. The size, location, and number of fibroids significantly influence the patients’ quality of life, making the precise determination of fibroid volume crucial for clinical decision-making [1].

Treatment of uterine fibroids encompasses various approaches, including medication, minimally invasive procedures such as uterine artery embolization (UAE), and surgical interventions like myomectomy and hysterectomy [2, 3]. Accurate assessment of fibroid size and number is essential for therapeutic decision-making. In clinical practice, a practical approach for estimating fibroid volume involves using the ellipsoid formula, calculated based on the three largest diameters of the tumor. The ellipsoid formula (D1 × D2 × D3 × 0.5233) allows for quick and easy volume estimation suitable for routine clinical application. Therefore, this method is commonly used in diagnostics and therapy planning in everyday practice [4]. However, fibroids are often not ellipsoidal in shape but instead have irregular forms, which can lead to errors in volume determination, especially when multiple fibroids are involved. Furthermore, variations among different fibroids and secondary degenerative changes in tumors can complicate accurate volume assessments. In contrast, manual segmentation using imaging software such as 3D Slicer or ITK-SNAP enables precise delineation of fibroid boundaries. This voxel-based measurement, accounting for the tumor’s actual shape, results in significantly more accurate volume estimates. Manual segmentation is highly time-consuming, requires specialized expertise, and is thus less commonly applied in clinical practice due to limitations in capacity and resources [5].

In the era of personalized medicine, where treatments are tailored to individual needs, accurate measurement of fibroid volume is of paramount importance. It is essential for diagnosis, treatment planning, and patient follow-up. Therefore, improving the precision of fibroid volume measurement is not only a scientific but also a clinically critical goal.

The aim of this study is to compare volume measurement using the ellipsoid formula applied in everyday practice with the gold-standard, ground truth manual segmentation method in terms of fibroid volume determination. Future investigations into such cases may help to identify factors that challenge the reliability of using the ellipsoid formula in clinical practice, suggesting the need for manual segmentation instead. We deemed it worth examining whether the more precise but significantly time-consuming manual segmentation technique is necessary to ensure quality in patient care. The research outcomes may contribute to refining clinical protocols and treatment guidelines, ultimately improving therapy and quality of life for patients with fibroids.

## Methods

Our study was approved by the Institutional Review Board (Semmelweis University Regional and Institutional Committee of Science and Research Ethics, SE-RKEB: 172/2022). As this was a retrospective study, the need for written informed patient consent was waived by the ethics committee. All procedures performed in this study involving human participants were in accordance with the ethical standards of the Declaration of Helsinki. All patient data were analyzed anonymously.

### Patient population

Consecutive patients who had uterine artery embolization at our Institution due to symptomatic uterine fibroids were retrospectively collected between May 2016 – September 2020. No rigorous patient selection criteria were applied, all patients were included who had pretreatment baseline MRI examinations at our Medical Imaging Centre. The MRI scans were retrieved from our institutional Picture Archiving and Communication System (PACS). A total number of 158 patients were identified. Out of these patients, 45 had to be excluded: 10 due to damaged DICOM files, 13 due to an extreme number of fibroids that cannot be delineated, 3 due to non-contourable fibroids (border not well-defined), 5 due to adenomyosis, and 14 due to two fibroids of similar size. Therefore, 113 patients were included in the analysis.

### MRI examination

The MRI examinations of the patients were performed at our Medical Imaging Centre, on a 1.5 T equipment (Philips Ingenia 1.5T, Philips Healthcare, Best, Netherlands) according to our routine contrast-enhanced pelvic MRI examination protocol. The MRI protocol included a T1W, a T2W, a T2W-SPAIR, as well as a contrast-enhanced T1W-SPIR sequence. The fibroids were contourable with the highest confidence on the axially reconstructed T2W-SPAIR sequence, which was available for all cases. Slice thickness and interslice gap were 4 mm and 1 mm, respectively. Therefore, we decided to use this sequence for further analysis. The flip angle was 90°, and the echo time was 80 s. The voxel spacing was 0.70 mm, and the reconstruction matrix was 400 × 400.

### Image postprocessing and segmentation

**Fig. 1:**
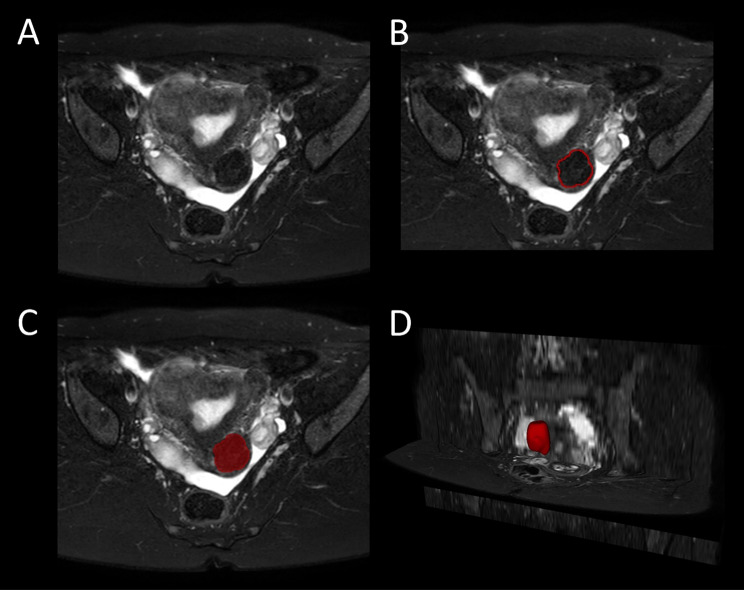
Illustration of manual, voxel-based segmentation using the 3D Slicer software. The T2W SPAIR axial sequences (**A**) were retrieved. The largest fibroids were manually delineated (**B**). The segmentation masks (**C**) were used for the reconstruction and calculation of the volumes (**D**)

The MRI scans were exported from the PACS system to a local institutional workstation. The DICOM format scans were converted to NIfTI file format eliminating all the metadata and preserving only the voxel intensity information. Currently, manual segmentation is the gold standard method [6]. The 3DSlicer software (slicer.org) was used for manual segmentation and further postprocessing [7]. The uterine fibroids were manually segmented slice-by-slice on the axial images (Fig. 1). If there were more fibroids with similar size, the fibroid with the biggest longest diameter was chosen. The cases were reviewed one-by-one to ensure that the same fibroid was selected as the largest using both diameter measurement-based and segmentation-based method. The diameter-based measurements were done on the same sequence and plane as the volumetry-based measurements (T2-weighted SPAIR axial MRI images). Slice thickness and gap was 4 mm and 1 mm, respectively. The longest diameter was measured in the axial plane, the second diameter was chosen as the biggest diameter perpendicular to the previously designated longest diameter. The third diameter was determined in the sagittal or coronal plane, perpendicular to the first and second diameters - measured from the uppermost to the lowermost point. The segmentation was performed based on the consensus opinion of two specialized board-certified radiologists with more than 15 and 20 years of experience in female pelvic MR imaging, respectively. The segmentation masks were then used for the calculation of the volume of uterine fibroids as reference standard.

### Diameter-based volume estimation by the ellipsoid formula

The diameter-based measurements were done on the same sequence and plane as the volumetry-based measurements. The longest diameter was measured in the axial plane, the second diameter was chosen as the biggest diameter perpendicular to the previously designated longest diameter. The third diameter was determined in the sagittal or coronal plane, perpendicular to the first and second diameters - measured from the uppermost to the lowermost point. The largest diameters (D1/D2/D3) of the fibroids were manually measured along the three standard planes, the volumes were then calculated by applying the formula of the ellipsoid (D1 × D2 × D3 × 0.5233) (group E). The volume of the calculated ellipsoid was then directly compared to the segmented volume of the fibroids (group S).

For the interobserver reproducibility of diameter measurements and diameter-based ellipsoid volume measurements, a second radiologist independently measured each fibroid blinded to the first’s results.

### Subgroup analysis according to the fibroids’ location

The fibroids were classified based on their location into submucosal, subserosal, intramural, and hybrid types. The segmented volumes, the volume differences between the two methods, and the correlation between the measurements were investigated for the subgroups.

### Statistical analysis

Data are reported as median (interquartile range – IQR) unless otherwise specified. The difference between the segmented volumes and the diameter-based calculated volumes was compared using the Wilcoxon signed-rank test. The agreement between the two observer’s diameter measures as well as the agreement in volume values between the ellipsoid-formula-based calculated vs. the segmented volumes were assessed by the intraclass correlation coefficient (ICC) analysis using two-way random effects, absolute agreement, single rater/measurement model, and the Bland-Altman plot analysis. To calculate the optimal cutoff where the difference between the two measurement methods starts to diverge, change-point detection was applied. Levene’s test was used to compare the variance between the two groups. The subgroup analysis for fibroid types was performed using the Kruskal-Wallis test with post-hoc Dunn’s test with Bonferroni correction.

## Results

Interobserver reliability analysis of diameter measurements confirmed excellent agreement in D1, D2, and D3 with ICCs of 0.978 [0.969–0.985], 0.978 [0.968–0.984], and 0.940 [0.907–0.960], *p* < 0.001, respectively. Similarly, the calculated volumes yielded excellent interobserver agreement (ICC = 0.971 [0.957–0.980]; *p* < 0.001).

Fibroids where the interobserver difference of diameter-based ellipsoidal volumes was > 30% were excluded, resulting in 99 patients in the final dataset reaching homogenous and reliable data.

Median volumes of Group S and Group E were 134.1 cm^3^ and 133.5 cm^3,^ respectively.

The IQR values of the fibroid volumes were 257.3 cm^3^ in Group S and 269.1 cm^3^ in Group E (0.25% average difference, *p* = 0.377). In 46 cases (46.5%), the values of the S group were larger, while in 53 fibroids (53.5%), the E group volumes were larger.

**Fig. 2:**
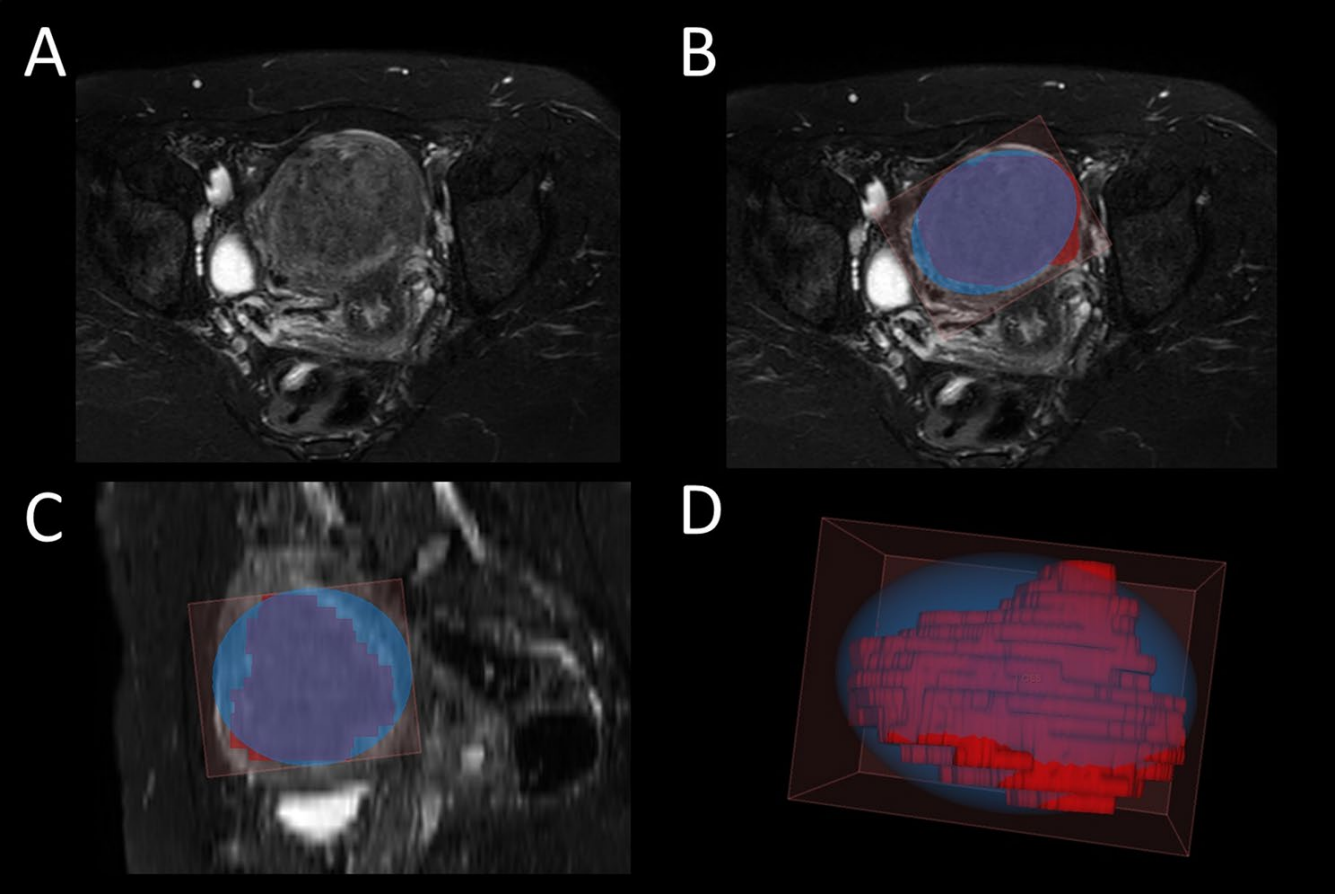
Overestimation of the volume of uterine fibroid by using the maximal diameters and the ellipsoid formula compared to the actual segmented volume. The axial reconstruction of the T2W SPAIR MRI sequence of a patient with multiple uterine fibroids (**A**). The manual segmentation of the largest fibroid (red) and its bounding ellipsoid (blue) on the axial plane (**B**), sagittal plane (**C**), and in three-dimensional reconstruction (**D**)

The shape of the segmented masks often deviated significantly from the ellipsoid form. In an illustrative case, we found that the manually segmented volume was 50.7 cm^3^ while the calculated volume was 79.4 cm^3^. In this case, the overestimation of the fibroid volume by using the ellipsoid formula was 28.7 cm^3^ (56.5%) (Fig. 2).

**Fig. 3:**
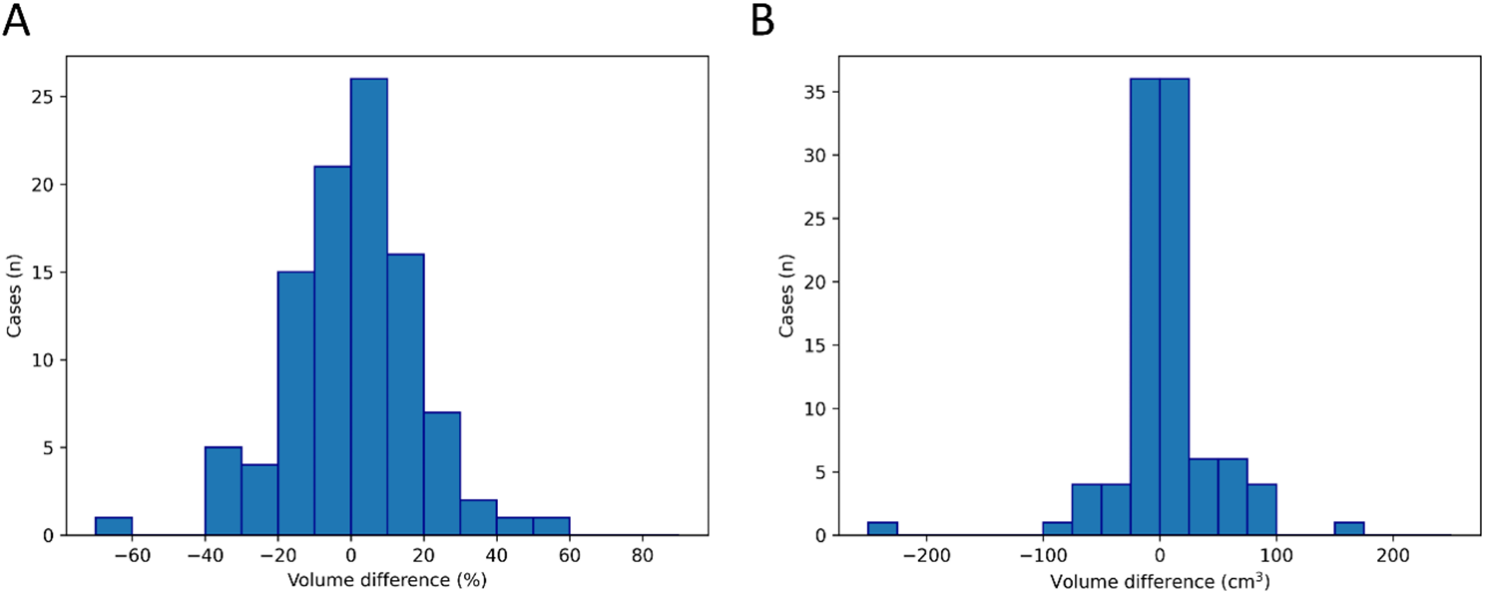
Histogram of measurement difference between the segmented fibroid volumes and the volumes estimated by the ellipsoid formula. **A** Difference in percentage calculated as volume measurement difference (cm3) ×100 / Segmented volume (ground truth). **B** Measurement difference in cm3. A negative value means that the volume calculated from the diameters is smaller than the segmented volume, suggesting an underestimation of the volume

Our histogram of measurement difference (%), i.e. measurement difference (ml) ×100 /segmented (ground truth) illustrates the distribution of the data and the outlier data as well. A negative value means that the volume calculated from the diameters is smaller than the segmented volume, suggesting an underestimation of the volume (Fig. 3).

**Fig. 4:**
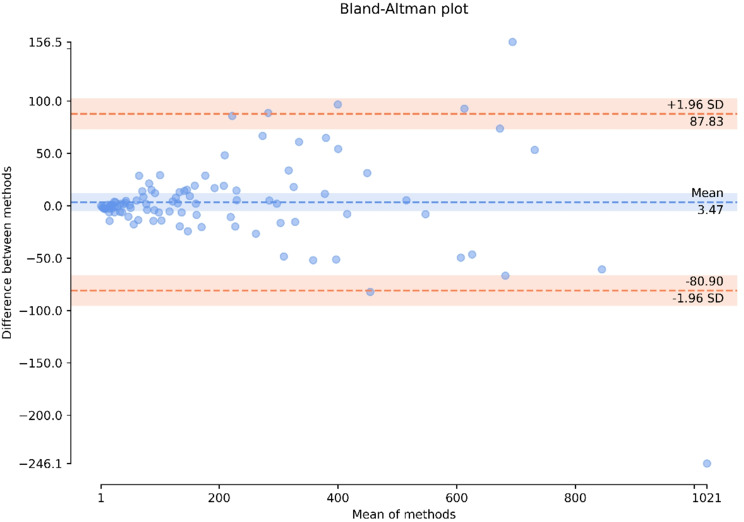
Bland Altman plot comparing the segmented (ground truth) volume (cm^3^) with the volume calculated from diameters using the ellipsoid formula

The Bland-Altman analysis showed that the mean difference between the two methods was 3.47 cm³, with data points evenly distributed around the mean, suggesting no substantial systematic bias in either volume measurement method. The limits of agreement (LoA), calculated as the mean difference ± 1.96 SD, ranged from − 80.90 cm³ to 87.83 cm³, indicating a slight tendency for the ellipsoid formula to overestimate volume. Additionally, the plot suggests that the discrepancy between the methods increases with fibroid size. Notably, in 4 cases, the differences exceeded the LoA indicating a larger difference between the methods than expected from a normal distribution (Fig. 4).

To define the optimal cutoff for ellipsoid-based volume estimation with a structural break in variance where the difference between the two measurement methods starts to diverge, change-point detection was applied resulting in a cut-off of 232.3 cm^3^ separating the cases into two groups (0.04, 11.50 cm^3^ vs. 8.37, 100.89 cm^3^ [median, IQR]) with significantly different variances (*p* < 0.0001).

**Fig. 5:**
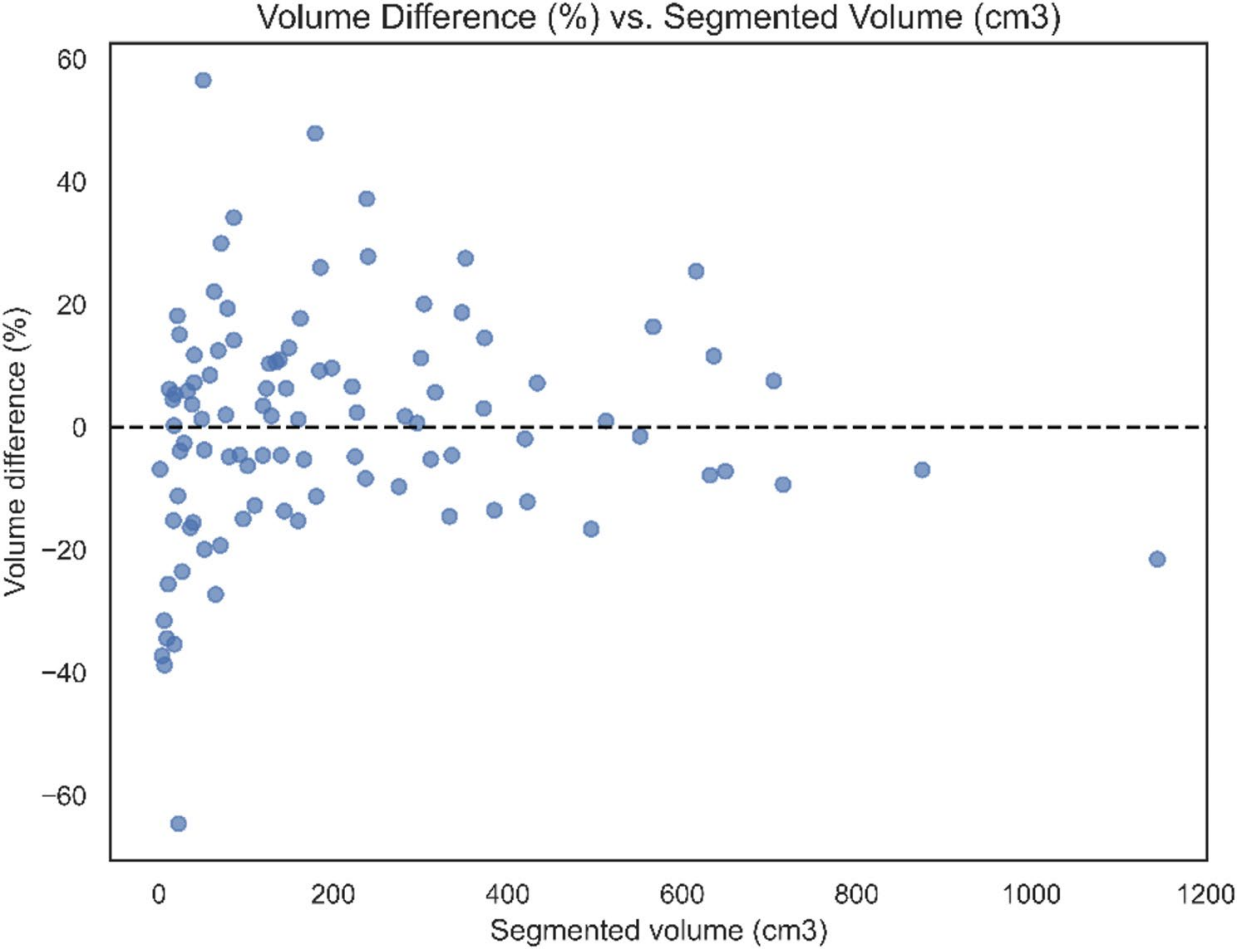
Association between the percentage volume difference and the volume of the fibroid

We also made a plot that illustrates the association between the percentage volume difference and the volume of the fibroid. The volume difference fell outside the ± 20% range in 21 cases (21.2%) and outside the ± 30% range in 10 cases (10.1%) (Fig. 5).

Subgroup assessment of fibroid types identified 29 subserosal, 21 submucosal, 43 intramural, and 6 hybrid-type fibroids. Although, the volumes of the submucosal fibroids were significantly smaller (48.91, 74.59 cm3) compared to the intramural (185.10, 271.03 cm3; *p* = 0.002; [median, IQR]), subserosal (134.05, 265.79 cm3; *p* = 0.015), and hybrid (286.41, 222.37; *p* = 0.012) types; the subgroups showed no significant differences in either absolute (cm3) or relative (%) volume measurement differences. The agreement between the two volume measurement methods was excellent for all subgroups with ICC of 0.985 [0.96–0.99] for submucosal, 0.977 [0.96–0.99] for intramural, 0.979 [0.96–0.99] for subserosal, and 0.960 [0.69–0.99] for hybrid types.

## Discussion

The main idea of the study is the comparison of the volume of uterine fibroids calculated using voxel-based segmentation and the ellipsoid formula. Currently, in clinical practice, the ellipsoid formula is used to determine the volume of fibroids. Its use is supported by its quick and simple applicability, and in our study as well as in the studies we examined, no significant differences were found between the volumes measured with voxel-based segmentation and those estimated using the ellipsoid formula. However, the relevance of our study lies in examining outlier data, as in the era of personalized medicine, it is not sufficient to treat based on statistical averages. Volume values can be crucial in establishing the final diagnosis, creating a treatment plan, and follow-up for different tumor types. Thus, in our study, we highlighted cases where the discrepancy exceeded 20% and 30%.

Figure 4 shows the Bland Altman plot comparing the segmented (ground truth) volume with the volume calculated from diameters using the ellipsoid formula. Fibroids above 232.3 cm^3^ with the ellipsoid formula have larger variability in volume difference between the two methods than the fibroids below 232.28 cm^3^; suggesting that segmentation for fibroids above 232.3 cm^3^ would be advisable for a more precise volume measurement.

In our study, we examined uterine fibroids. However, the majority of the previous studies we found in connection with our research have examined prostate cancer. In our study, there was no significant difference between the ellipsoid formula and voxel-based volume calculations for the entire group. Hamzaoui et al. reported in 2022 on the MRI examination of 40 patients with prostate cancer before treatment, finding that the prostate volumes measured using the ellipsoid formula and manual segmentation were well reproducible but with a slight overestimation [8]. Stanzione et al.‘s retrospective analysis from 2021 found a high correlation between the ellipsoid formula and manual segmentation in 195 MRI examinations, using ITK-SNAP software [9]. In two recent studies on the prostate, both suggested that the deep learning model or semi-automated segmentation should be preferred to the ellipsoid formula [10, 11]. Beside prostate, another recent study on cystic jaw lesions’ volume came to the conclusion that computer-aided volume assessment confers an advantage over the ellipsoid-formula based volume data [12]. In our study, special attention was paid to outlier data, with 21 cases (21.2%) showing > 20% and 10 cases (10.1%) showing > 30% discrepancies; the largest difference was approximately 56.5% (Fig. 3). In the above-mentioned studies, however, there was no mention of outlier data [8, 9]. Similarly, in a retrospective study by Youn et al. in 2023, where 456 patients with both transrectal ultrasound and MRI findings from two university hospitals were included, excellent agreement was found between the ellipsoid formula and the gold standard (segmentation). It was noted that prostate volume was overestimated on MRI, while underestimated on ultrasound. Bland-Altman plots clearly show a significant number of outliers, but these are not discussed in detail in the article [13]. Lin et al., based on prostate MRI examinations of 76 patients, also concluded that there was a high agreement between the segmentation and the volumes determined by the ellipsoid formula. Additionally, the authors documented outlier cases. The outlier patient data were mainly present due to post-transurethral resection and irregular hyperplastic nodules. Regarding this, data were provided for only one patient, who was excluded. The prostate volume calculated by the segmentation method was 47.2 ml, while the value calculated based on the ellipsoid volume formula was 33.6 ml (approximately 29% volume underestimation) [14]. Outlier data are also not discussed in the two recent papers [10, 11].

The results should be evaluated considering the limiting factors of the research. Our research is limited by being a single-center, retrospective study.

## Conclusions

The ellipsoid formula-based and the voxel-based volume calculation showed no significant difference for the group as a whole. However, there was a difference of > 20% in 21.2% of cases and > 30% in 10.1% of cases; the largest difference was approximately 56.5%. The difference between the two methods showed significantly larger variability above 232.3 cm^3^ suggesting the potential need for manual segmentation for more precise volume assessment.

In the era of personalized medicine, it is not only the average difference between the two methods that need to be considered but also cases where there is a substantial (e.g. 20% or 30%) difference in the results (outlier data) should be highlighted, as these may change the treatment plan in individual cases. This methodology should also be tested for other tumor type volume calculations also.

## Data Availability

Hereby I confirm that all data generated or analysed during this study are included in this published article. Viktor Bérczi, MD, PhD, DMSc, EBIR, CIRSE Distinguished Fellowprofessor, deputy-headMedical Imaging Clinic, Department of Radiology, Semmelweis University1082 Üllői út 78/a, Budapest, Hungarye-mail: berczi@hotmail.com; berczi.viktor@semmelweis.humobile: +36-20-825-8091.
